# Serological based monitoring of a cohort of patients with chronic Chagas disease treated with benznidazole in a highly endemic area of northern Argentina

**DOI:** 10.1590/0074-02760160006

**Published:** 2016-06

**Authors:** Leticia L Niborski, Vanina Grippo, Sonia O Lafón, Gabriela Levitus, Facundo García-Bournissen, Juan C Ramirez, Juan M Burgos, Margarita Bisio, Natalia A Juiz, Vilma Ayala, María Coppede, Verónica Herrera, Crescencia López, Ana Contreras, Karina A Gómez, Juan C Elean, Hugo D Mujica, Alejandro G Schijman, Mariano J Levin, Silvia A Longhi

**Affiliations:** 1 Instituto de Investigaciones en Ingeniería Genética y Biología Molecular, Consejo Nacional de Investigaciones Científicas y Tecnológicas, Buenos Aires, Argentina; 2 Hospital de Niños Ricardo Gutiérrez, Servicio de Parasitología y Chagas, Buenos Aires, Argentina; 3 Centro Asistencial Cáritas Diocesana, Añatuya, Santiago del Estero, Argentina; 4 Hospital Zonal Añatuya, Santiago del Estero, Argentina

**Keywords:** Trypanosoma cruzi, chronic Chagas disease, benznidazole treatment, serological follow-up, adverse effects

## Abstract

This study aimed to evaluate well-documented diagnostic antigens, named B13, 1F8 and JL7 recombinant proteins, as potential markers of seroconversion in treated chagasic patients. Prospective study, involving 203 patients treated with benznidazole, was conducted from endemic areas of northern Argentina. Follow-up was possible in 107 out of them and blood samples were taken for serology and PCR assays before and 2, 3, 6, 12, 24 and 36 months after treatment initiation. Reactivity against *Trypanosoma cruzi* lysate and recombinant antigens was measured by ELISA. The rate of decrease of antibody titers showed nonlinear kinetics with an abrupt drop within the first three months after initiation of treatment for all studied antigens, followed by a plateau displaying a low decay until the end of follow-up. At this point, anti-B13, anti-1F8 and anti-JL7 titers were relatively close to the cut-off line, while anti-*T. cruzi* antibodies still remained positive. At baseline, 60.8% (45/74) of analysed patients tested positive for parasite DNA by PCR and during the follow-up period in 34 out of 45 positive samples (75.5%) could not be detected *T. cruzi* DNA. Our results suggest that these antigens might be useful as early markers for monitoring antiparasitic treatment in chronic Chagas disease.

Chagas disease or American trypanosomiasis, caused by the parasite *Trypanosoma cruzi*, is endemic in much of the Americas, from the southern United States to Argentina and Chile. It is the fourth most common tropical disease, after malaria, tuberculosis and schistosomiasis, currently affecting 7-6 million people (WHO 2015). The risk of infection with *T. cruzi* is directly related to poverty, but due to migrations, several cases have been reported throughout the world (Lescure et al. 2008, Muñoz et al. 2009, Jackson et al. 2010).

In Argentina, it is estimated as many as 1.5 million patients have Chagas disease and 2.2 million people in risk of *T. cruzi* infection (WHO 2015). The endemic area covers the north of the country where the conditions, such as high levels of poverty and social exclusion, low population density, mostly rural, subsistence economy, and a weak health system, favor not only *T. cruzi* infection but also for the development of this disease.

Once the individual acquires the parasite, the infection starts with an acute phase, followed by a chronic stage which includes asymptomatic and symptomatic cases, with cardiac, digestive manifestations or mixed patterns (WHO 2015). Up to now, the available treatment is based on two drugs: nifurtimox and benznidazole (BNZ). Chemotherapy against *T. cruzi* infection is strongly recommended for all cases during the acute stage, in children under 15 years old and reactivated infections in immunocompromised patients (Bianchi et al. 2015), but its effectiveness during the chronic stages is still under revision (Cançado 2002, Viotti et al. 2006, 2014). Some studies suggest that BNZ for asymptomatic or early symptomatic cases may improve parasite clearance rates (de Andrade et al. 1996, Sosa-Estani et al. 1998). In 1999, a panel of experts reached the consensus that patients with chronic Chagas disease should be treated with an anti-*T. cruzi* medication (PAHO 1999). From this recommendation, many studies are being conducted. Thus, results from a multicenter, placebo-controlled trial involving BNZ for the treatment of Chagas cardiomyopathy showed that the drug significantly diminished serum parasite detection, but did not improve cardiac clinical manifestation (Morillo et al. 2015). In parallel, another trial with long-term follow-up in adult patients, is being conducted in Argentina to evaluate whether BNZ treatment change the evolution of chronic Chagas disease (Riarte 2013). Other randomised clinical studies, with shorter follow-up periods, based on the safety and efficacy of new drugs such as posaconazole, studied this drug alone or in combination with BNZ (Molina et al. 2014, and STOP CHAGAS clinical trial, Identifier: NCT01377480).

After treatment, the criterion of cure in chronic Chagas disease is the persistence of negative parasitological and serological results (Porrás et al. 2015). Unfortunately, *T. cruzi* lysate antibody seroconversion occurs several years after antiparasitic therapy in most individuals, while parasitological methods are consistently negative (Guedes et al. 2011, Machado-de-Assis et al. 2012). Thus, the identification of early markers of seronegative conversion is an important and imperative step to evaluate Chagas disease treatment.

The aim of this study was to evaluate if well-known serological markers could have an early predictive value and be useful to monitor drug therapy response, by measuring antibody (Ab) levels over time in a cohort of patients with chronic Chagas disease treated with BNZ. For this purpose, we selected the recombinant proteins named B13, 1F8 and JL7, because they are recognised by most chronic chagasic patients and are currently used in commercial kits for diagnosis (Umezawa et al. 1999, 2003, Ponce et al. 2005).

## SUBJECTS, MATERIALS AND METHODS

*Patients and study design* - Prospective study was carried out from years 2000-2004 in Añatuya, a city located in a highly Chagas disease-endemic area in the Province of Santiago del Estero - Argentina. Three hundred and twelve *T. cruzi*-infected adults volunteers aged 15-55 years were recruited and 270 patients in the asymptomatic or cardiac chronic phase of Chagas disease were eligible.

Inclusion criteria were positive diagnostic for Chagas disease by commercial ELISA and indirect hemagglutination assays, according to Argentina’s National Guidelines and the World Health Organization (WHO) recommendations. Exclusion criteria included the visceral damage, presence of systemic arterial hypertension, liver or kidney failure, severe cardiac lesions associated with other cardiac diseases, pregnancy, lactation, alcoholism and hypersensitivity to the drug. BNZ was given three times daily over 60 days at a total dose 5 mg/kg/day (100 mg/tablet: Radanil, Roche). During treatment, patients were subjected to clinical examination to evaluate side effects.

Blood samples were collected at the following instances: T0, before treatment; T1, two months; T2, three months; T3, six months; T4, one year; T5, two years and T6, three years after treatment onset, respectively.

The research protocol followed the tenets of the Declaration of Helsinki and was approved by the Local Medical Ethics Committees named Comité Institucional de Ética de Investigación en Salud - Ministerio de Salud y Desarrollo Social de Santiago del Estero and Comité de Ética del Hospital Zonal de Añatuya Monseñor Gottau. Before and during the beginning of this study, the area was under triatominae vector surveillance by official government sanitary agents and Mundo Sano Private Foundation (i.e., they undertook regular insecticidal actions against infestations). All enrolled adult patients gave written informed consent and the parent or guardian of the child participants provided informed consent on their behalf after the nature of the study was explained.

*DNA extraction and amplification* - Blood samples were mixed with an equal volume of 6 M guanidine HCl/0.2 M EDTA buffer pH: 8.0. Guanidine-EDTA blood (GEB) was heated for 15 min in boiling water and total DNA was purified from 500 µL GEB with phenol-chloroform-isoamyl alcohol (25:24:1, V/V) as previously reported (Schijman et al. 2003). The 330-bp variable regions of the *T. cruzi* kinetoplastid minicircle genome was amplified with 121 [5′-AAATAATGTACGG G(T/G)GAGATGCATGA-3′] and 122 (5′-GGTTCGATTGGGGTTGGTGTAATATA-3′) primers by conventional PCR as previously described (Schijman et al. 2003).

*Parasite lysate and recombinant proteins* - Parasite extracts were obtained from *T. cruzi* epimastigotes CL-Brener strain DTU Tc VI (Zingales et al. 2009), as previously described (Gómez et al. 2001).

B13, 1F8 and JL7 were expressed as GST fusion proteins and purified by affinity chromatography on glutathione-agarose beads as previously described (Umezawa et al. 1999). Protein contents were quantified by Bradford reagent, and all antigens were maintained at -80ºC until used.

*Enzyme-linked immunosorbent assay (ELISA)* - In-house ELISAs were carried out as described previously (Longhi et al. 2012). Briefly, microwells (Costar Inc., Corning, NY) were coated overnight at 4ºC with *T. cruzi* lysate (20 μg/mL) or B13, 1F8 and JL7 recombinant proteins (5 μg/mL) in 50 μL of 0.05 M carbonate-bicarbonate buffer (pH 9.6). The plates were washed thrice with washing buffer (PBS containing 0.1% Tween-20; PBS-T) and then blocked with PBS-T plus 5% non-fat dry milk for 1 h at 37ºC. Sera diluted 1:200 in PBS-T with 1% non-fat dry milk were incubated in duplicate for 2 h at 37ºC. To avoid test variability, serum samples (from T0-T6) from a given patient were assayed on the same plate and sera from eight healthy individuals were also loaded on each plate to determine cut-off value.

After the plates were washed three times, 50 μL of alkaline phosphatase (for *T. cruzi* lysate) or peroxidase (for recombinant proteins) conjugated anti-human polyvalent immunoglobulins diluted 1:3000 in PBS-T containing 1% non-fat dry milk, was dispensed into each well and incubated 1 h at 37ºC. The reaction was developed with p-nitrophenyl phosphate (for alkaline phosphatase) or 3, 3’, 5, 5’ - tetramethylbenzidine substrate (for peroxidase) and read at 415 nm or 450 nm, respectively.

In parallel, some serum samples were also analysed by commercial Chagatest ELISA recombinant v4.0 (Wiener Laboratory, Rosario, Argentina) according to manufacturer’s instruction.

*Statistical analysis* - Chi-square test for the analysis of drug side-effects were performed. Longitudinal panel data analysis was used to assess the change in Ab levels over time. Longitudinal data was analysed using generalised linear mixed effects modelling, as implemented in R software 2.10.0 version (R Core Team 2013), with random effects specified at the level of the individual. Normal distribution of dependent variables was evaluated with Shapiro-Wilks test. Variables not normally distributed were log-transformed before analysis and log-normality confirmed with Shapiro-Wilks test for the transformed variables. Estimation of 95% confidence intervals (95% CI) for generalised linear mixed effects regressions was done by Markov Chain Monte Carlo, as implemented in WinBUGS and R (R Core Team 2013). Statistical significance was assumed at p < 0.05 or 95% CI.

## RESULTS AND DISCUSSION

Several recombinant antigens are used for diagnosis of Chagas disease (Afranchino et al. 1989, dos Santos et al. 1992, Krieger et al. 1992, Umezawa et al. 1999, 2003, Di Noia et al. 2002, Camussone et al. 2009, De Marchi et al. 2011), but none of them has been tested as early markers of cure after anti-parasitic treatment. With this idea in mind, we decided to determine the level of antibodies against three antigens, namely B13, 1F8 and JL7, in a cohort of patients treated with BNZ by an in-house ELISA method. These antigens correspond to repeat tandem proteins of *T. cruzi* and have been well described in other works as immunodominant antigen in Chagas disease (Umezawa et al. 1999, 2003, Longhi et al. 2012).

**Fig. 1:**
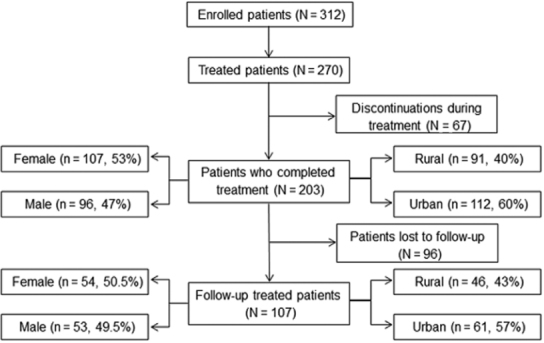
: flow diagram of patient enrollment, treatment and follow-up.

Flowchart of enrollment, treatment and follow-up is schematised in Fig. 1. Sixty seven out of 270 enrolled patients (24.8%) interrupted treatment, because of death (2/67, 2.9%), pregnancy (4/67, 5.9%), fail to correctly follow medical prescription or adverse effects (24/67, 35.8%) and voluntary abandonment (37/67, 55.2%). Although 203 completed drug therapy, only three or more follow-up samples could be obtained in 107 treated patients, especially due to difficulties of patients to attend to hospital during three years of drug monitoring. The number of samples analysed from T0-T6 is shown in Table. It is important to mention that the group of patients analysed was representative of the total cohort that completed treatment with similar rates of females/males and rural/urban residents (Fig. 1). Moreover, mean age of patients who completed treatment (34.5 years, N = 203) or follow-up (35.7 years, N = 107) was also comparable, with a higher proportion of patients ranged in age from 25-44 years (65%; 132/203 and 68%; 73/107, respectively).

**TABLE:** Number of samples analysed during the benznidazole follow-up

	Time^a^	N^b^
T0	0	107
T1	2	104
T2	3	106
T3	6	104
T4	12	73
T5	24	65
T6	36	60

^a^Time in months from treatment onset; ^b^Number of serum samples.

As it has been previously reported (Viotti et al. 2009, Pinazo et al. 2010) typical side effects, generally mild and transient, were observed in 67 out of 203 patients who complete treatment. The most common side effect proved cutaneous hypersensitivity (29/203, 14.3%) such as pruritus, allergy and exanthema, followed by digestive disorders (18/203, 8.8%) as epigastric pain, dyspepsia and heartburn. In addition, 11 out of 203 patients (5.4%) presented headache, nauseas or vomiting, while nine patients suffered arthralgia and myalgia (4.4%). The appearance of more than one side effect was only observed in 12 out of 203 patients (5.9%). The incidence of adverse reactions was not statistically associated to age, sex, or place of residence -urban or rural areas- (*Chi-square test*, p = 0.43, p = 0.26, p = 0.96, respectively). It is important to highlight that a high number of patients, who developed cutaneous hypersensitivity, worked in brick factories exposed to heat and sunlight.

Parasitological experiments were performed in 74 out of 107 patients. At baseline, 60.8% (45/74) had positive results for *T. cruzi* PCR, whereas during monitoring period, consistently negative PCR results were documented in 34/45 (75.5%). Similar data have been reported by Morillo et al. (2015) in BENEFIT trial, showing that *T. cruzi* DNA was not detected in 73% of Argentinian and Bolivian patients at the end of follow-up. However, CHAGASAZOL trial reported that 94% of patients treated with BNZ tested negative by PCR (Molina et al. 2014). These differences could be related to the risk of exposure to the parasite, because patients included in CHAGASAZOL trial have been living in Barcelona, Spain - a non-endemic area - during the whole study. On the contrary, although the area was under triatominae vector surveillance, the study population in BENEFIT trial and in our work belonged to endemic regions, where there is a real risk of exposure to reinfections, due to population movement to areas without vector control.

**Fig. 2:**
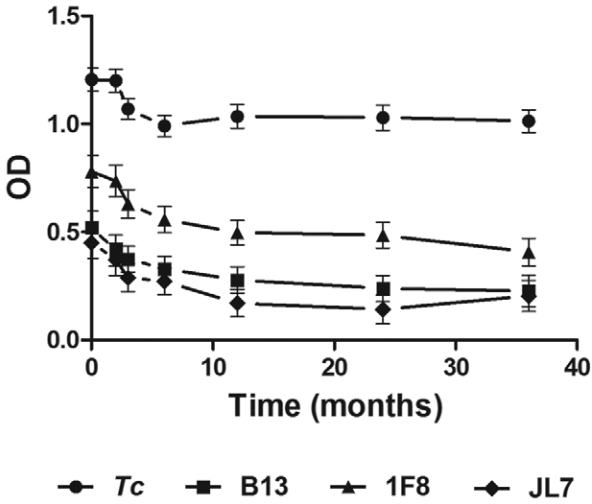
: antibody levels. ELISA plates were coated with *Trypanosoma cruzi* lysate, or B13, 1F8 and JL7 recombinant proteins in buffer carbonate. Serum samples were diluted 1/200 and tested in duplicate. Results are expressed as optical density (OD) means for each antigen ± standard error of the means (SEM). Cut-off values for *T. cruzi*, B13, 1F8 and JL7 recombinant proteins, were 0.276, 0.110, 0.367 and 0.150, respectively.

After analysing serologic response against *T. cruzi* lysate or recombinant proteins by linear mixed effects modelling, we observed an apparent biphasic curve for all antigens, with an initial rapid decline within the first three months followed by a plateau with a slight decay for the remaining 36 months of follow-up (Fig. 2). The decrease in Ab titers against *T. cruzi* lysate over time was -0.0075 optical density (OD)/month (95% CI -0.0060 to -0.0090]), with a significant effect of time for each patient (Supplementary figure). Similar results were obtained for Ab anti-B13, anti-1F8 and anti-JL7 levels with a slope of -0.0032 OD/month for B13 (95% CI [-0.0010 to-0.0074], -0.0041 OD/month for 1F8 (95% CI [-0.0001 to -0.0083]) and -0.0032 OD/month for JL7 (95% CI [-0.0009 to -0.0075]), with significant effect for each patient as a function of time (Supplementary figure). A statistically significant faster rate of decline was observed for Ab titers against *T. cruzi* lysate in comparison to anti-B13, anti-1F8 and anti-JL7 (p < 0.01 for all comparisons, linear mixed effects model analysis). Indeed, results showed that the OD mean values for recombinant proteins declined progressively close to the cut-off values at the end of the follow-up, while those for *T. cruzi* lysate remained positive (Fig. 2). This observation could be explained by the fact that sera reactivity at T0 is higher to *T. cruzi* lysate than with recombinant proteins. This data is in accordance with results presented by others, who observed a low rate of seronegative conversion of anti-*T. cruzi* response detected by conventional serological test (Sosa-Estani et al. 1998, 2, 2009, Viotti et al. 2011, , Machado-de-Assis et al. 2013).

**Fig. 3:**
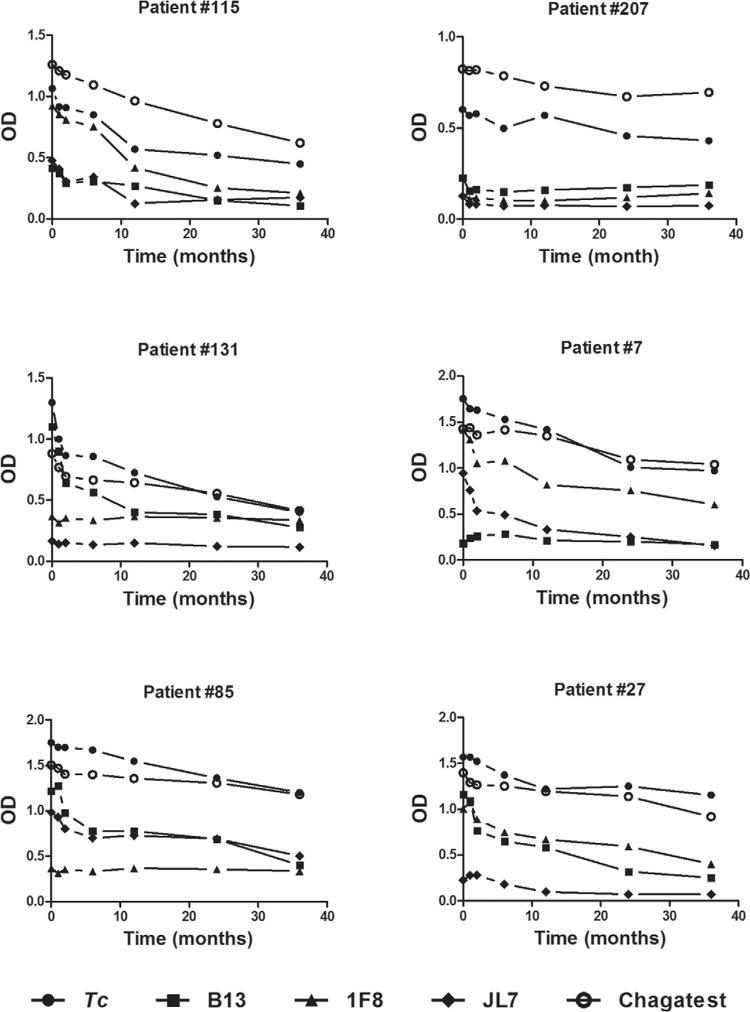
: variation in optical density (OD) by the ELISA as a function of time. Results from six patients, representing typical pattern response against *Trypanosoma cruzi* lysate, B13, 1F8 and JL7 recombinant proteins, are schematised. Chagatest ELISA recombinant v4.0 results were included in comparison of in-house ELISAs. Cut-off values for *T. cruzi*, Chagatest, B13, 1F8 and JL7 recombinant proteins, were 0.276, 0.126, 0.110, 0.367 and 0.150, respectively.

It is important to highlight that most sera presented reactivity against *T. cruzi* lysate and the recombinant proteins, as for example the Patient #115 (Fig. 3), but some patients did not developed detectable Ab against all the recombinant antigens. As shown in Fig. 3, serum from Patient #207 did not react with the three studied recombinant antigens and along with serum from Patient #7 were the only two samples that showed low reactivity against B13. Likewise five patients had a similar pattern response as Patient #131, seven patients as Patient #85 and 12 patients as Patient #27 without reactivity to 1F8 and/or JL7 (Fig. 3). In this cohort of patients, the percentage of reactivity against B13, 1F8 and JL7 was 98, 89 and 82, respectively. Based on these finding, we suggest that no single recombinant protein would be useful for the assessment of therapeutic response in all patients, and reactivity against the three antigens should be tested individually for future treatment evaluation.

In parallel for comparison purpose, sera reactivity was determined by a commercial Chagatest ELISA recombinant. As shown in Fig. 3, a similar pattern response to *T. cruzi* lysate was obtained, with a slight decrease in Ab levels, but without showing a seroconversion at end of follow-up, while as mentioned above, anti-B13, anti-1F8 and anti-JL7 Ab declined progressively close to the cut-off values at the end of follow-up. Finally, we observed along to monitoring that Patients #115, #131, #7, #85 and #27 showed decay in Ab response, especially against to recombinant antigens (Fig. 3) and also tested negative for *T. cruzi* DNA by PCR. On the contrary, Patient #207, who had no changes in reactivity against *T. cruzi* lysate (Fig. 3), PCR assays remained positive during the follow-up.

Throughout the years, there have been major advances in the control of *T. cruzi* transmission due to vector control by insecticide spraying, but little has been achieved in terms of treatment of infected people. The fact that most of those infected individuals are from the poorer strata of society diminishes the interest in the development of new drugs by the pharmaceutical companies, but it is one of the priorities for WHO in the control of Chagas disease. Therefore, therapy efficacy or drug success necessarily requires validate an accurate method to evaluate the course of the disease (Pinazo et al. 2014).

As mentioned above, positive prognosis after anti-parasite treatment often is made by serological and/or parasitological subsequent examinations with consistently negative results in time (Porrás et al. 2015). Nonconventional serological methods, including a multiplex format or ELISA with recombinant proteins, have been developed to provide fast, easy to measure, responses to etiological treatments (Cooley et al. 2008, Fernández-Villegas et al. 2011, Fabbro et al. 2013). However, there are no consensuses about which is the gold standard of post-treatment evaluation protocols to establish cure in a reasonably short time (Urbina 2015).

Overall, our results showed that none of the patients showed a seronegative conversion for anti-*T. cruzi* response during the follow-up, but a significant decay in the Ab titer against recombinant antigens was observed in the majority of the patients with chronic Chagas disease three years after the end of specific drug treatment.

Since, the level of Ab against recombinant proteins B13, 1F8 and JL7 decreased to cut-off line earlier than Ab against *T. cruzi* lysate and these reduction were not statistically associated to age, sex, or place of residence, results suggest that these antigens would be good candidates as early markers of serological response to evaluate drug success. Although the main limitations of our study were the non-randomised design, and absence of a placebo control group due to bioethics considerations of the Local Medical Ethics Committee, our findings encourage further evaluation of these recombinant antigens in randomised and controlled trial with trypanocidal drugs.
